# Toxic Epidermal Necrolysis after COVID-19 mRNA-1237 Vaccination

**DOI:** 10.1155/2023/8855665

**Published:** 2023-11-14

**Authors:** Nicole Hehr, Benjamin P. Davis

**Affiliations:** ^1^Carver College of Medicine, Iowa City, USA; ^2^Division of Immunology, Department of Internal Medicine, University of Iowa, Iowa City, IA, USA

## Abstract

This letter illustrates a case of toxic epidermal necrolysis (TEN) after COVID-19 mRNA-1273 vaccination, which corresponds with the existing published data and contributes detailed knowledge of TEN reaction after vaccination. Interestingly, the reaction started at the site of vaccination and the patient went on to tolerate a major excipient of the vaccine suggesting the reaction may be associated with the mRNA itself or is triggered by the immunostimulatory action of the vaccine.

## 1. Introduction

A 30-year-old Caucasian female with a past medical history of exercise induced asthma, polycystic ovarian syndrome, obesity, migraines, hypermobility, irritable bowel syndrome, depression, anxiety, and attention deficit hyperactivity disorder was admitted to an outside hospital with blistering skin lesions covering 30%–40% of her body for 12 days after receipt of a COVID-19 messenger ribonucleic acid (mRNA)-1273 vaccine. She reported allergies to topiramate (respiratory distress), cephalosporins (gastrointestinal upset), and benzalkonium chloride (rash). Her home medications included ibuprofen as needed, modafinil, aripiprazole, buspirone, oral cholecalciferol, citalopram, cyanocobalamin, ferrous sulfate, hydroxyzine, linaclotide, mono-linyah, and triamcinolone ointment. She had been on all medications a minimum of 10 weeks prior to presentation.

**Figure 1:**
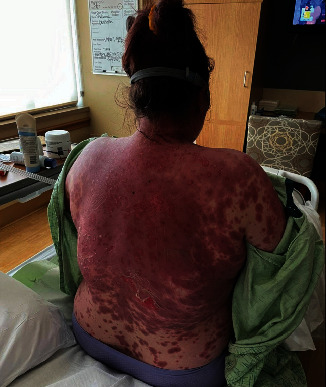
Targetoid lesions and skin sloughing in toxic epidermal necrolysis.

The patient and her husband both received the mRNA-1273 vaccine on the same day. That night, she noticed swelling in her fingers that prompted her to take ibuprofen, which she had taken as needed prior to that day. Two days later, she noticed a cluster of skin lesions around the injection site on her left arm. These symptoms prompted her to visit her primary care provider, who prescribed prednisone, cetirizine, and hydroxyzine. By Day 12 after injection, her rash continued to spread and she was seen at an outside dermatology clinic. Presumed diagnosis at the dermatology clinic was erythema multiforme (EM) versus Stevens–Johnson syndrome (SJS) due to classic targetoid lesion appearance of her rash (Figure 1). Biopsy showed “interface dermatitis with dyskeratotic epidermis, intact stratum corneum, and focal re-epithelialization” consistent with EM. That same day, she was sent to the emergency department again for admission. On admission, her rash was described as “confluent diffuse erythematous targetoid rash” on the face and chest with bullae present in the axillae and back. She was also noted to have some skin sloughing on the back, scattered lesions on her abdomen and legs, and desquamating oral lesions with crusting around her eyes and injection of the conjunctive (Figure 1). During this admission, she tested negative for COVID-19 and was treated with methylprednisolone 125 mcg every 6 hr planned for 5 days. After she failed to improve, she was given IV flebogamma 5%. The patient reported the use of benzalkonium chloride spray on her back at some time during her admission that led to worsening of the skin on her back with more blistering and sloughing. As she continued to worsen, our hospital was contacted for admission to the burn unit for care.

Transfer occurred on Day 16 after vaccination. Dermatology recommended hydroxyzine 25 mg nightly, cetirizine 10 mg twice daily, and a dexamethasone 0.1 mg/mL rinse for mouth lesions with an antiseptic diluted chlorohexidine rinse. Over the course of her admission to our hospital, total body surface area (TBSA) coverage was estimated at 30%–40%, suggesting progression to toxic epidermal necrolysis (TEN) [1]. Psychiatry was also consulted for management of the patient's psychiatric medications and her modafinil and aripiprazole were stopped on suspicion of being a cause of her TEN. Psychiatry restarted her on buspirone and citalopram. The patient was discharged home on Day 21. Of note, our patient reported a colonoscopy with prep (polyethylene glycol (PEG) 3350) that occurred after her TEN reaction without incident.

## 2. Conclusion

Here, we report SJS/TEN that seems very likely to be triggered by a mRNA vaccine as the rash originated from site injection. To date, we are aware of only three reported cases of EM/SJS/TEN spectrum reactions after COVID-19 mRNA vaccines [2, 3]. Of the cases published, the general timeline to symptoms appears to be 3–5 days [2, 3], similar to our patient.

There were originally concerns that our patient's aripiprazole (started 7 months before reaction), modafinil (started 3 months before reaction), or ibuprofen (taken night of injection and used previously) could have caused her reaction. All three of these medications have been reported to be associated with SJS/TEN. Our patient continues to avoid these medications. However, given that these medications were all started at least 10 weeks before her COVID-19 mRNA vaccination or were taken previously, our suspicion is lower that these were contributory. Since the start of the rash began around the site of injection, the vaccine, at the very least, was likely an initiator of the reaction.

Regarding vaccine ingredients as potential triggers of the reaction, the ingredients in the vaccine include mRNA, producing the encoded S (spike) glycoprotein from the SARS-CoV-2 virus, lipids in the form of PEG 2000 and dimyristoyl glycerol, 1,2- distearoyl-sn-glycero-3-phosphocholine, cholesterol, tromethamine (and tromethamine HCl), acetic acid, sodium acetate trihydrate, and sucrose [4]. PEG is reported to have various anaphylactic type reactions, including death in severe cases, but PEG has not been reported to be associated directly or individually with SJS/TEN [5]. However, there are very rare case reports of SJS/TEN occurring with anti-tumor necrosis factor-PEG and recombinant erythropoietin-PEG [1, 6]. Our patient's history of having had PEG 3500 after this incident without reaction decreases suspicion that this is the ingredient that triggered her reaction.

This letter illustrates a case of TEN after COVID-19 mRNA-1273 vaccination, which corresponds with the existing data and contributes detailed knowledge of TEN reaction after vaccination and highlights the importance of awareness of the possibility of SJS/TEN reactions to mRNA vaccines.

## Data Availability

All data are contained in the patient's electronic medical record.

## Abbreviations

mRNA: Messenger ribonucleic acid
SJS: Stevens–Johnson syndrome
TEN: Toxic epidermal necrolysis
EM: Erythema multiforme
PEG: Polyethylene glycol.
